# Research hotspots and trends in malignant hyperthermia due to anesthesia from a global perspective: a bibliometric analysis from 1975 to 2024

**DOI:** 10.1186/s13023-025-03766-5

**Published:** 2025-07-01

**Authors:** Hou-Ming Kan, Chi Wang, Jin-Zhao Huang, Meng-Liu Sha, Hong-Yan Ni, Li-Ping Chen, Xiao-Tong Ding

**Affiliations:** 1 School of Pharmacy, Faculty of Medicine, Macau University of Science and Technology, Macau SAR, China; 2 School of Anesthesiology, Xuzhou Medical University, Xuzhou, China; 3 Department of Anesthesiology and Perioperative Medicine, Suqian Branch of Jiangsu Provincial People’s Hospital, Suqian, China; 4 Department of Pain, Affiliated Hospital of Xuzhou Medical University, Xuzhou, China; 5 School of Nursing, Chinese Academy of Medical Sciences & Peking Union Medical College, 33 Ba Dachu Road, Shijingshan District, Beijing, 100144 China

**Keywords:** Malignant hyperthermia, Anesthesia, Dantrolene, Ryanodne receptor, Bibliometric analysis

## Abstract

**Background:**

Malignant hyperthermia is a rare and highly lethal anesthesia emergency. We summarized the published articles on malignant hyperthermia to understand its development trends and provided meaningful recommendations for reducing the mortality rate.

**Methods:**

The Web of Science core, PubMed Central and Scopus databases were searched from 1975 to 2024 to identify articles related to malignant hyperthermia. The retrieved documents were exported as full-text records via CiteSpace 6.4. R1 and R (BiblioShiny packages) were used to measure and visually analyze the number of articles, journals, keywords, and references.

**Results:**

According to the bibliometric analysis, 1473 publications were published by research teams across 60 countries and regions. In 1473 publications, advanced economies accounted for 87.78% (*n* = 1303), while emerging and developing economies accounted for 12.22% (*n* = 170). In the past 40 years, advanced economies have dominated the field of MH research. The research on MH in emerging and developing economies have been rapidly increasing since 2010. Emerging and developing economies were paying more attention to MH diagnosis and the specific drug dantrolene. The most prolific country was the United States (*n* = 439, centrality = 0.55). The University of California System (*n* = 44, centrality = 0.04) had the highest number of articles. The journal ANESTHESIOLOGY was cocited 731 times and had the highest number of cocitations. From the timeline of keyword appearance, attention was devoted to dantrolene and succinylcholine in the 1980s. Since the 1990s, more research has focused on MH susceptibility and ryanodine receptors. Recently, amide anesthetics and spinal anesthesia-induced MH have received more attention.

**Conclusions:**

We revealed the evolutionary path of MH research through bibliometric analysis. Owing to the lack of dantrolene and cooperation mechanisms in developing countries, the mortality rate caused by MH is still high. The government and pharmaceutical companies should collaborate to assist the availability and accessibility of dantrolene in developing countries.

**Supplementary Information:**

The online version contains supplementary material available at 10.1186/s13023-025-03766-5.

## Introduction

Malignant hyperthermia (MH) is a severe reaction caused by the inhalation of volatile anesthetics and depolarizing neuromuscular blocking drugs in genetically susceptible patients [1]. MH is a rare drug-related genetic disease associated with pathogenic variations in the Ryr1, CACNA1S, or STAC3 genes [2]. The typical clinical manifestations of MH include rhabdomyolysis, hypercapnia, and sudden high temperatures [3]. The morbidity of MH during anesthesia ranges from 1/30,000 to 1/10,000 [4]. Without being exposed to anesthetics or inducing drugs, humans who are prone to malignant hyperthermia do not experience any phenotypic alterations. Consequently, a greater number of individuals may be at risk for MH. However, the incidence of MH in children is far greater than that in adults and significantly greater in men than in women [5, 6]. At present, the international gold standard for the diagnosis of people with suspected malignant hyperthermia is the extracorporeal coffee morphine‒fluorane contracture [7]. Owing to the use of dantrolene and the routine detection of end-tidal carbon dioxide (ETCO_2_), the mortality rate of MH has decreased from 80% to less than 10% [8]. However, this situation has not improved in developing countries, where the mortality rate of MH is still relatively high owing to the lack of medication and standardized rescue procedures. Although some reviews and guidelines exist to describe the disease characteristics of MH, these articles are based on regional or specific time points. Bibliometrics is an effective tool that helps researchers visualize the evolution of published literature [9]. Han et al. [10] conducted a bibliometric analysis of the top 100 cited literatures in 2023, highlighting the characteristics of the most influential studies on MH. However, most of the literatures in this study focuses on developed countries. Currently, there is a lack of research on MH from a global perspective. The mortality rate of MH is relatively high, especially in developing countries. We summarized the published articles on MH to understand its development trends and provided meaningful recommendations for reducing the mortality rate. The analytical dimensions of bibliometrics include the year of publication, country and region, institution, journal, author, keywords, and cocitations. Our primary goal is to understand the global research trends and hot topics related to MH by analyzing relevant literature from 1975 to 2024. Our secondary goal is to compare the current research status of MH between developed and developing countries and provide meaningful recommendations for reducing the mortality rate.

## Methods

### Data collection and strategy for data retrieval

**Fig. 1:**
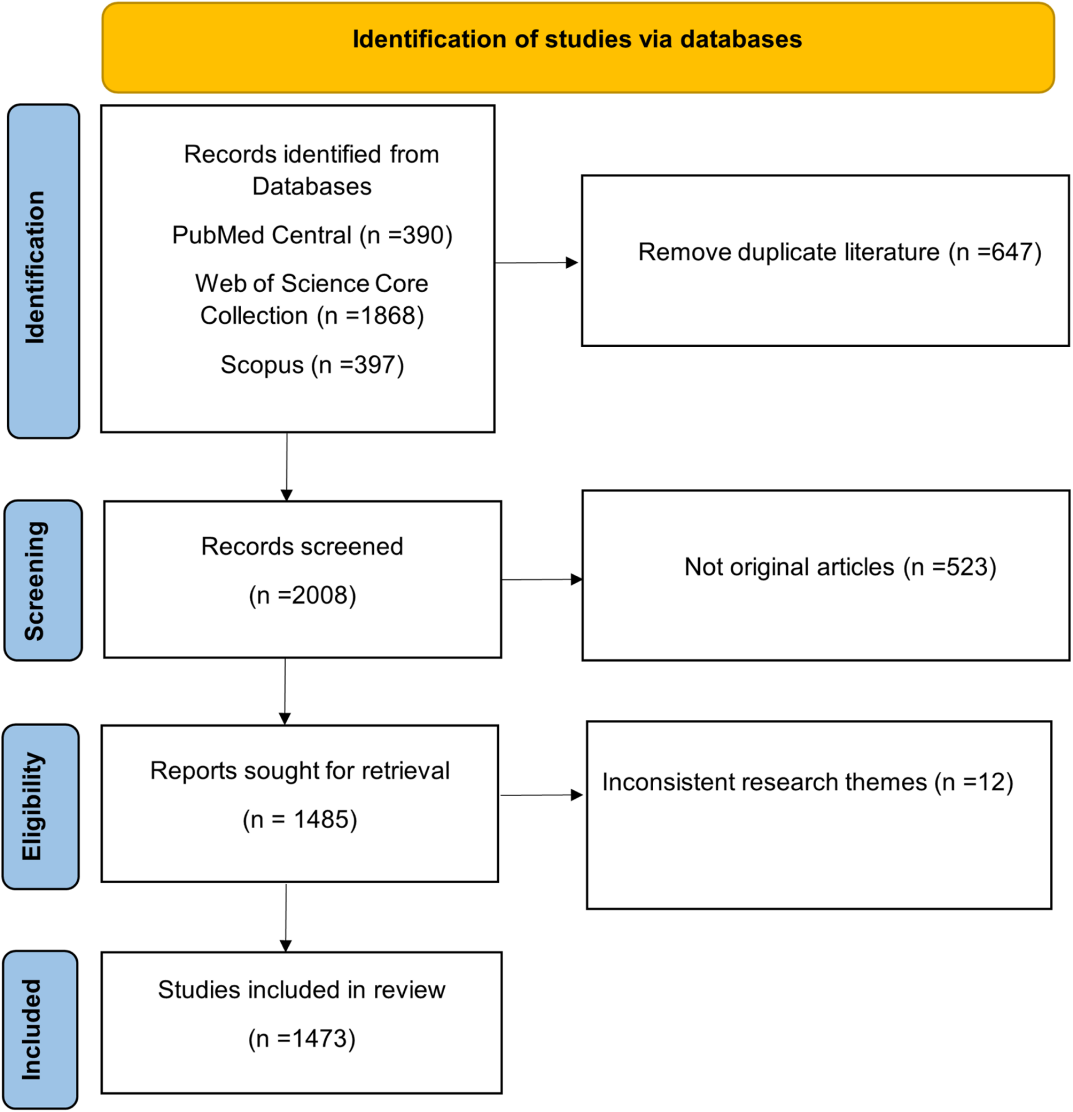
Literature search and inclusion flowchart. The search was completed on 1 January 2025. Through deduplication and screening of the literature, 1473 articles were ultimately included for analysis

We searched the Web of Science (WoS) Core Collection, PubMed Central (PMC) and Scopus databases to retrieved studies. We downloaded the entire records and cited references of the literature. This study used malignant hyperthermia as the MeSH keyword (https://www.ncbi.nlm.nih.gov/mesh) for the literature search. We screened the literature based on the following criteria: (1) the search terms and strategies are shown in Table 1; (2) the document type was an article; (3) the publication date ranged from 1 January 1975 to 31 December 2024; and (4) the language of publication was not limited. Non-English articles identified in the screening process were included if they provided an English abstract with sufficient data for analysis. The search was completed on 1 January 2025. We first used NoteExpress (Aegean, Beijing) for preliminary deduplication, and then performed manual deduplication. After deduplication and screening, 1473 articles were ultimately included for analysis (Fig. 1). We divided the research subjects into two categories based on the 2024 International Monetary Fund (IMF), namely advanced economies and emerging & developing economies. Then we conducted subgroup analysis on the current research status of MH in these two categories of economies.

**Table 1:** Search terms and strategies

Databases	Search strategy
Scopus	TITLE-ABS-KEY(“Anesthesia Related Hyperthermia”) OR TITLE-ABS-KEY(“Malignant Hyperpyrexia”) OR TITLE-ABS-KEY(“Hyperthermia of Anesthesia”) OR TITLE-ABS-KEY(“Anesthesia Hyperthermia”) OR TITLE-ABS-KEY(“Malignant Hyperpyrexia”)
PMC	Search (Anesthesia Related Hyperthermia[MeSH Terms]) OR (Malignant Hyperpyrexia [MeSH Terms]) OR (Hyperthermia of Anesthesia [MeSH Terms]) OR (Anesthesia Hyperthermia [MeSH Terms]) OR (Malignant Hyperpyrexia[MeSH Tems]) OR (Anesthesia Related Hyperthermia [Title]) OR (Malignant Hyperpyrexia [Title]) OR (Hyperthermia of Anesthesia [Title]) OR (Anesthesia Hyperthermia [Title]) OR (Malignant Hyperpyrexia [Title]) OR (Anesthesia Related Hyperthermia [Abstract]) OR (Malignant Hyperpyrexia [Abstract]) OR (Hyperthermia of Anesthesia [Abstract]) OR (Anesthesia Hyperthermia[Abstract]) OR (Malignant Hyperpyrexia [Abstract])
WoS Core Collection	TS=(Anesthesia Related Hyperthermia) OR TS=(Malignant Hyperpyrexia) OR TS=(Hyperthermia of Anesthesia) OR TS=(Anesthesia Hyperthermia) OR TS=(Malignant Hyperpyrexia)

Note: Search on 1 January 2025. Retrieval time range: 1975 to 2024. Abbreviations: TS, subjects; PMC, PubMed Central; WoS, Web of Science

### Data analysis and network mapping

The data were imported into CiteSpace V6.4 R1. (https://citespace.podia.com/) and R (BiblioShiny packages), which can be used to analyze the potential information contained in complex data and to visualize the structure, correlation, and centrality through visual means.

Co-occurrence analysis refers to counting the frequency of occurrence of multiple phrases in the same article to determine their proximity and obtain hot spots and future trends in the discipline. Co-citation analysis is used to discover the primary literature and knowledge in this research field [11]. The results provide information on the past, present, and future dimensions of the research field. Microsoft Office Excel 2021 and Origin 2024 were used to analyze publication trends. The pathogenesis model diagram was plotted using Adobe Illustrator 2020 and BioRender online tool.

In the visualization map, each metric represents a node, and the diameter of the circle represents how often the label appears in the co-occurrence analysis. The color of a circle is determined by the cluster of categories to which it belongs. The connection between nodes represents the association of the corresponding node, and the strength of the association between nodes is expressed in line width [12].

## Results

### Coauthors’ country and institution analysis

**Fig. 2:**
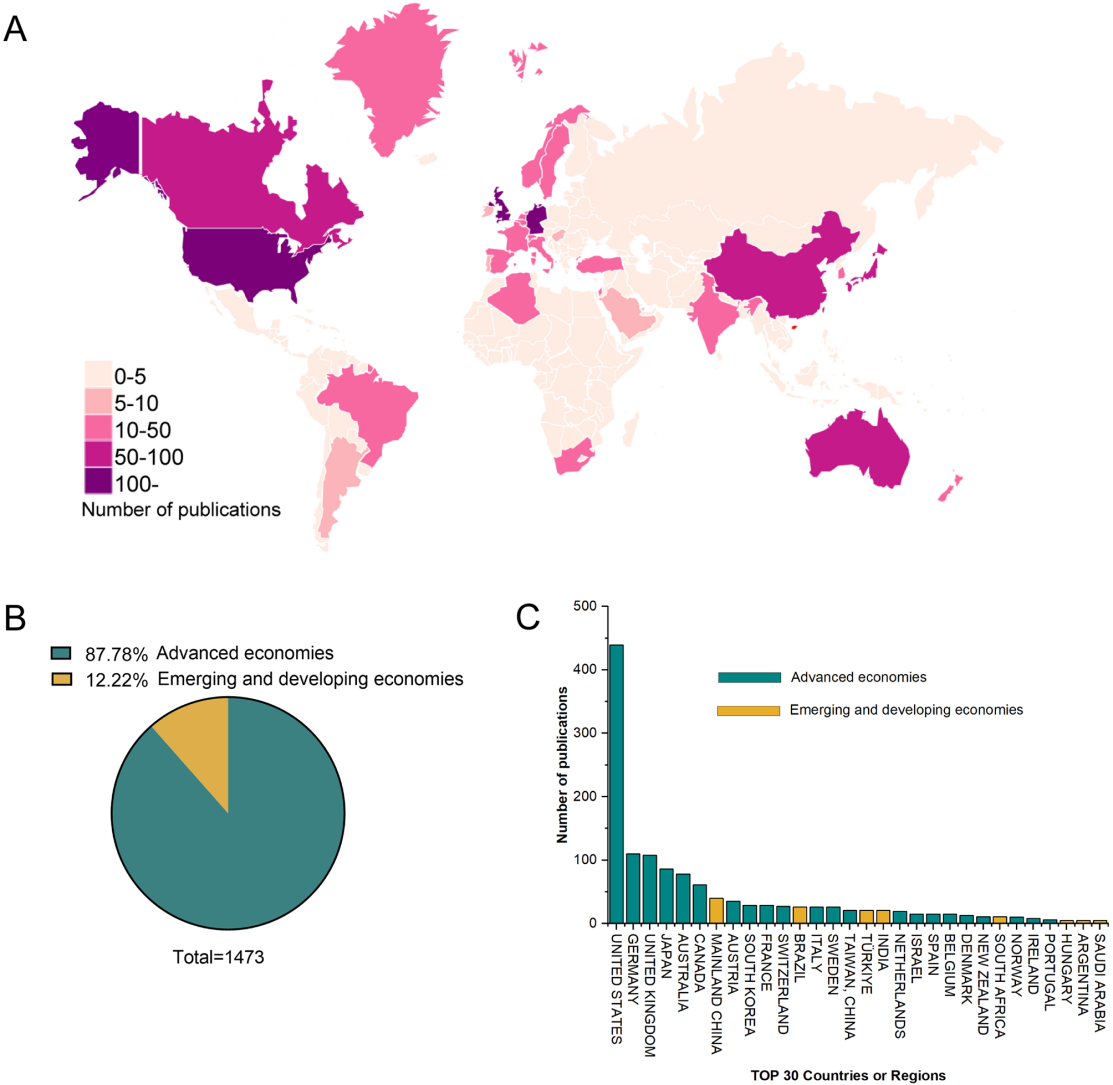
Coauthors’ country and institution analysis. (**A**) A bibliometric study revealed that research teams from 60 different nations and regions published 1473 publications. Research is comparatively active in North America, Western Europe, and East Asia. (**B**) Percentage of publications from two different economies. Advanced economies account for 87.78%, while emerging and developing economies account for 12.22%. (**C**) The top 30 countries or regions in terms of publication quantity

**Fig. 3:**
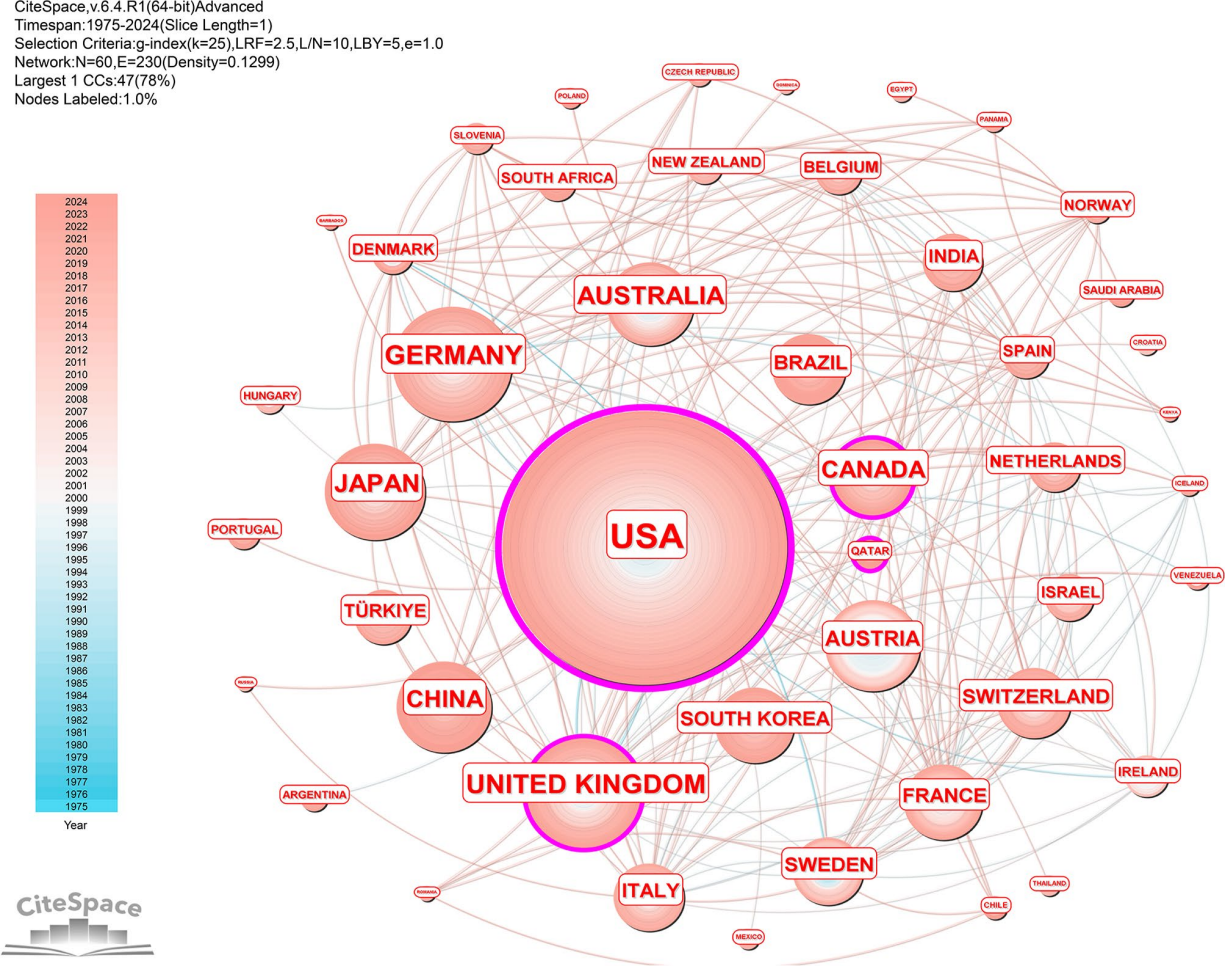
Network map of the country analysis of the coauthors. The most prolific country was the United States (*n* = 439, centrality = 0.55). The three countries that ranked after the United States were Germany (*n* = 110, centrality = 0.09), the United Kingdom (*n* = 108, centrality = 0.16), and Japan (*n* = 86, centrality = 0.02)

CiteSpace’s bibliometric study revealed that research teams from 60 different nations and regions published 1473 publications. North America, Western Europe, and East Asia were the regions that were most active in this field of research (Fig. 2A). The most prolific country was the United States (*n* = 439, centrality = 0.55), followed by Germany (*n* = 110, centrality = 0.09), the United Kingdom (*n* = 108, centrality = 0.16), and Japan (*n* = 86, centrality = 0.02). In 1473 publications, advanced economies accounted for 87.78% (*n* = 1303), while emerging and developing economies accounted for 12.22% (*n* = 170) (Fig. 2B). The top 30 countries or regions in terms of publication quantity can be seen in Fig. 2C. In terms of centrality and citations, the United States ranked significantly higher than other nations did (Fig. 3). The top 5 productive countries or regions in 2 categories are listed in Table 2.

**Table 2:** The top 5 productive countries or regions in 2 categories

Rank	Advanced economies		Emerging and developing economies	
	Country/region	Publications	Country/region	Publications
1	United States	439	Mainland China	40
2	Germany	110	Brazil	26
3	United Kingdom	108	Türkiye	21
4	Japan	86	India	21
5	Australia	78	South Africa	11

Author visualization analysis revealed three main author networks that are relatively influential in this field. The three author networks are Denborough from Australian National University in Australia, Sessler from Univ Calif San Francisco in the United States, and Ellis from St James’s University Hospital in the United Kingdom (Supplementary Fig. 1). Most of the publications are from the University of California System (*n* = 44, centrality = 0.04) (Supplementary Fig. 2). The top 5 institutions with publications in 2 categories are displayed in Table 3.

**Table 3:** The top 5 institutions with publications in 2 categories

Rank	Advanced economies		Emerging and developing economies	
	Institution	Publications	Institution	Publications
1	University of California System	44	University of São Paulo	16
2	Australian National University	29	University of Witwatersrand	11
3	Harvard University	25	Sichuan University	9
4	Mayo Clinic	22	University of Cape Town	5
5	University of Toronto	19	Peking Union Medical College	5

### Annual global publication outputs on malignant hyperthermia

**Fig. 4:**
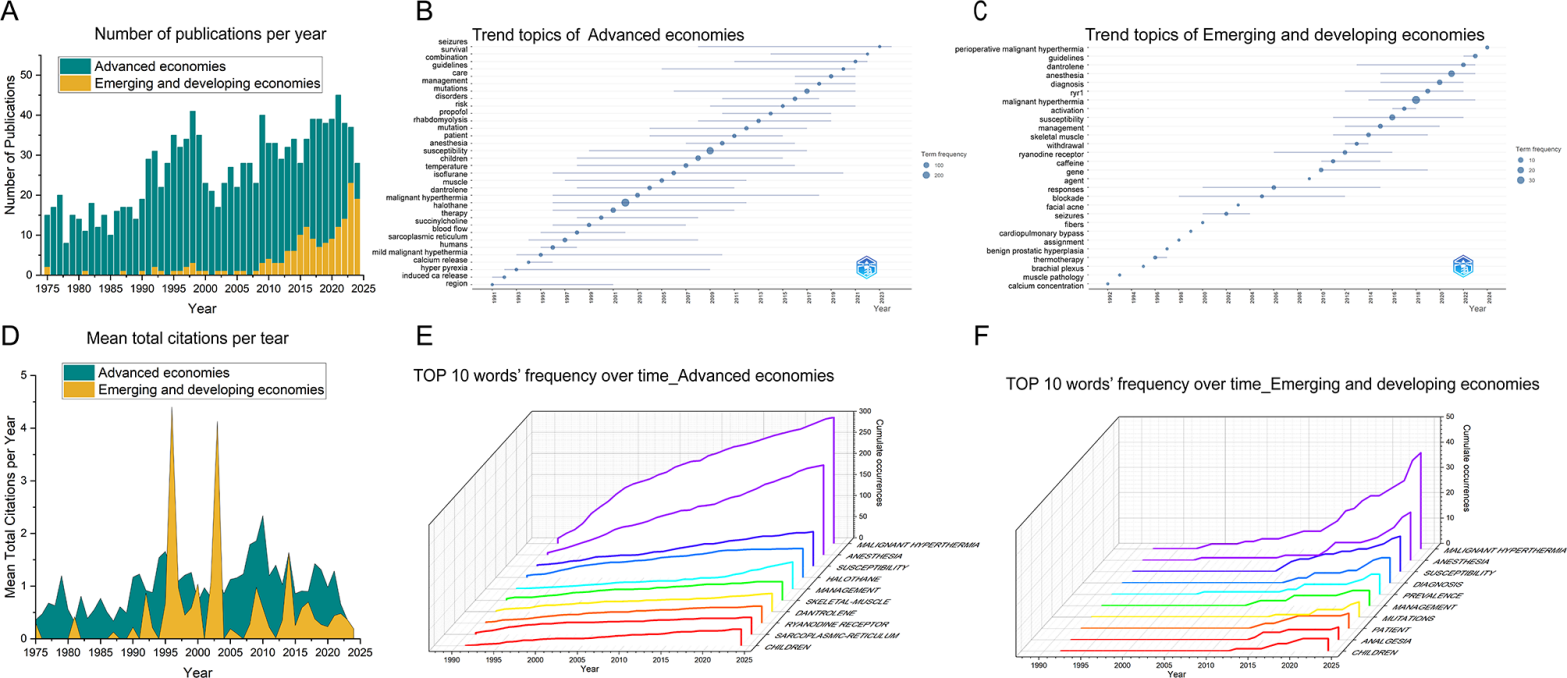
Subgroup analysis of advanced economies and emerging and developing economies. (**A**) Annual publication trends. In the past 40 years, advanced economies have dominated the field of MH research. The research on MH in emerging and developing economies have been rapidly increasing since 2010. (**B**-**C**) The annual trend of keywords changes in two different economies. Both economies have been paying attention to the ‘guidelines’ since 2020. The popularity of ‘dantrolene’ in emerging and developing economies has been sustained in recent years. (**D**) Mean Total Citations per Year. The Mean total citations per year of literatures from developed economies is mostly higher than that of emerging and developing economies, except for 1996 and 2003. (**E**-**F**) Top 10 words’ frequency over time. The top three keywords in both groups are consistent, namely malignant hyperthermia, anesthesia, and susceptibility

The annual publication trend of MH is shown in Fig. 4A. A total of 1473 publications were included in this study. Owing to MH being a relatively rare clinical phenomenon, the number of articles published on this topic worldwide is not very high. In the past 40 years, advanced economies have dominated the field of MH research. The research on MH in emerging and developing economies have been rapidly increasing since 2010.

### The annual trend of keywords changes in two different economies

Count the frequency of keywords appearing in different years and observe the trend of research topics in two different economies over time (Fig. 4B-C). Both economies have been paying attention to the ‘guidelines’ since 2020. Developed economies have paid attention to some special symptoms of MH, such as seizures. Emerging and developing economies pay more attention to the specific drug dantrolene for treating MH.

### Mean total citations per year

Mean total citations per year is the average number of citations per year since the publication of a literature. $\:\text{M}\text{e}\text{a}\text{n}\:\text{t}\text{o}\text{t}\text{a}\text{l}\:\text{c}\text{i}\text{t}\text{a}\text{t}\text{i}\text{o}\text{n}\text{s}\:\text{p}\text{e}\text{r}\:\text{y}\text{e}\text{a}\text{r}=\frac{\text{T}\text{o}\text{t}\text{a}\text{l}\:\text{c}\text{i}\text{t}\text{a}\text{t}\text{i}\text{o}\text{n}\text{s}}{\text{Y}\text{e}\text{a}\text{r}\text{s}\:\text{s}\text{i}\text{n}\text{c}\text{e}\:\text{p}\text{u}\text{b}\text{l}\text{i}\text{c}\text{a}\text{t}\text{i}\text{o}\text{n}}$. The Mean total citations per year of literatures from developed economies was mostly higher than that of emerging and developing economies, except for 1996 and 2003 (Fig. 4D).

### Top 10 words’ frequency over time

From the perspective of growth trends, developed economies tend to have a flat growth trend, while emerging and developing economies are growing rapidly (Fig. 4E-F). The top three keywords in both groups are consistent, namely malignant hyperthermia, anesthesia, and susceptibility. Developed economies pay more attention to the triggering factors and pathogenesis of MH. Emerging and developing economies pay more attention to MH diagnosis.

### Analysis of cocited journals

**Fig. 5:**
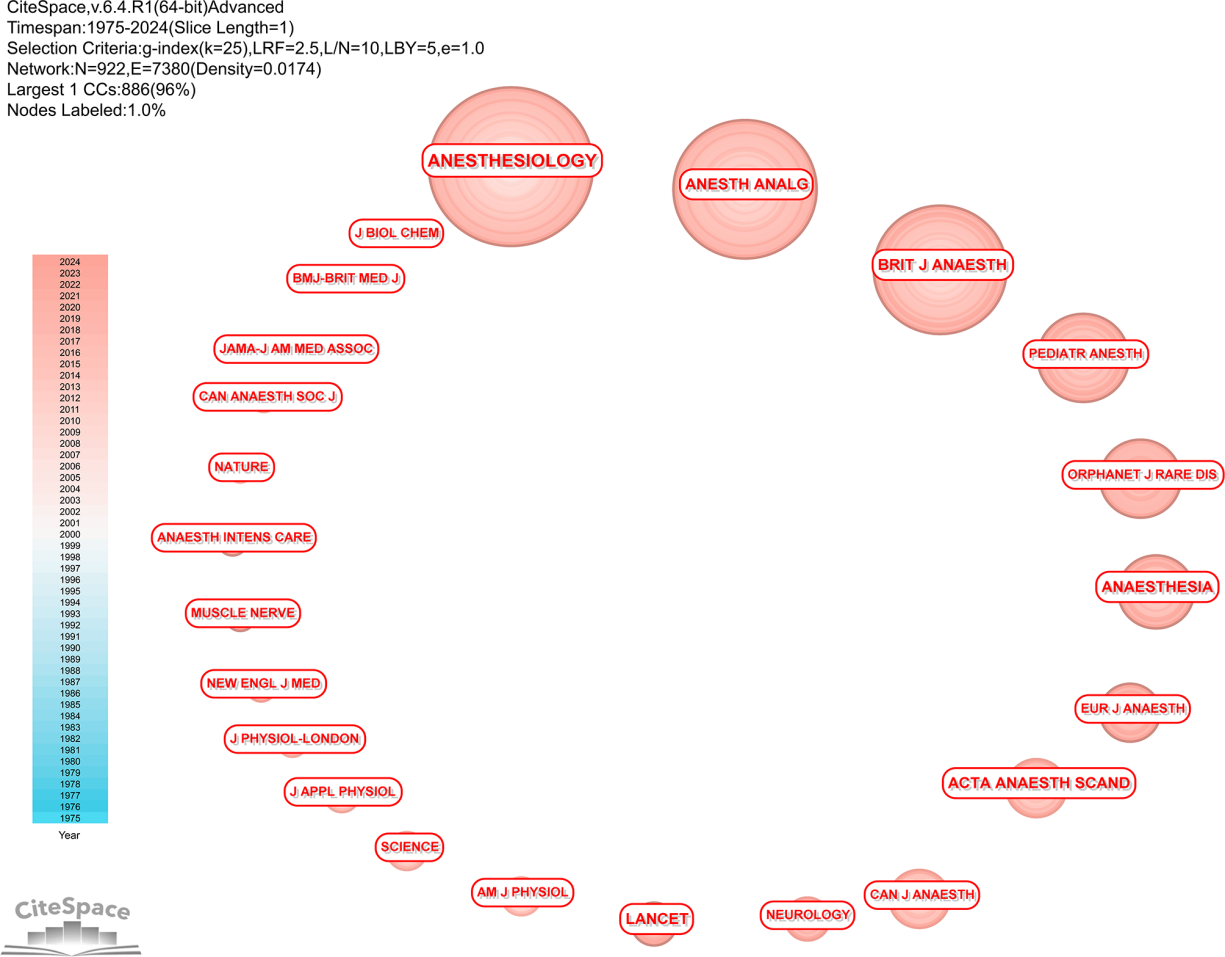
Cocitation analysis of journals. The journal ANESTHESIOLOGY was cocited 731 times and had the highest number of cocitations

The top 10 journals with the most significant cocitation counts are presented in Table 4. The journal *ANESTHESIOLOGY* was cocited 731 times and had the highest cocitation. More details can be found in Fig. 5.

**Table 4:** Top 10 cocitation journals in the MH field

Rank	Count	Centrality	Journals	IF(2023)	Country affiliation
1	731	0.00	Anesthesiology	9.1	United States
2	611	0.03	Anesth Analg	4.6	United States
3	611	0.00	Brit J Anaesth	9.1	United Kingdom
4	338	0.03	Lancet	98.4	United Kingdom
5	311	0.07	Anaesthesia	7.5	United Kingdom
6	265	0.06	Acta Anaesth Scand	1.9	Denmark
7	201	0.08	New Engl J Med	96.2	United States
8	194	0.03	Can J Anesth	3.4	Canada
9	156	0.04	Nature	50.5	United Kingdom
10	142	0.04	Pediatric Anesthesia	1.7	United Kingdom

### Document cocitation analysis

**Fig. 6:**
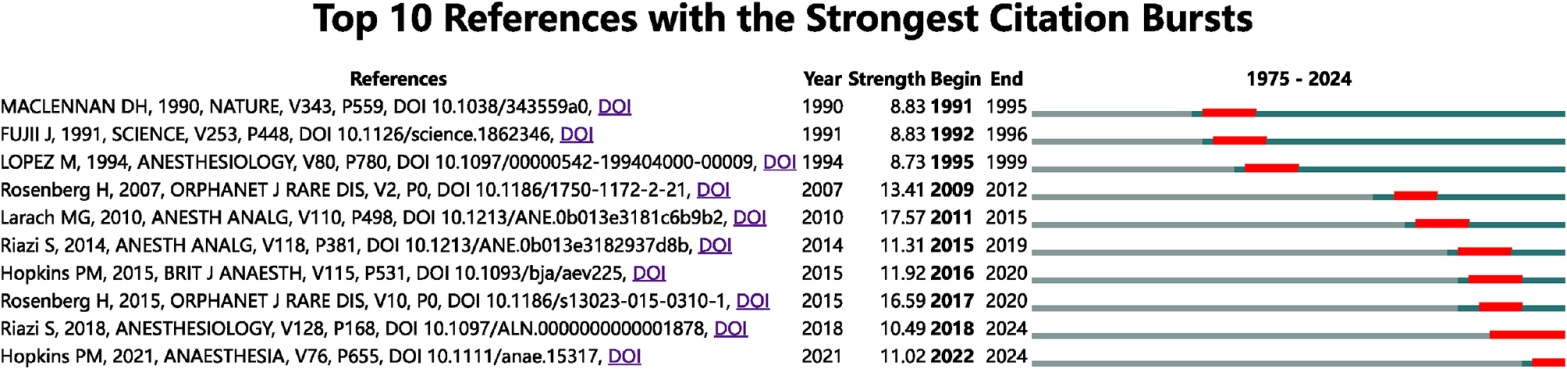
The top 10 references with the strongest citation bursts. The strongest cocitation bursts (17.57) were found in the reference Larach MG. Among the ten references, Riazi S and Hopkins PM had citation bursts that lasted until 2024, indicating that they remain the hotspots of contemporary research references

Figure 6 displays the top 10 references [6, 7, 13–20] with the strongest citation bursts. The strongest cocitation bursts (17.57) were found in the reference Larach MG [17]. Among the ten references, Riazi S [19] and Hopkins PM [20] had citation bursts that lasted until 2024, indicating that they remain the hotspots of contemporary research references.

### Keywords analysis

A total of 934 keywords were retrieved from 1473 documents. The high-frequency keywords are *MH* (count = 606, centrality = 0.62), *anesthesia* (count = 239, centrality = 0.59), *susceptibility* (count = 84, centrality = 0.06), *halothane* (count = 69, centrality = 0.07), *management* (count = 56, centrality = 0.06), and *dantrolene* (count = 48, centrality = 0.07). From the timeline of keyword appearance, attention was devoted to dantrolene and succinylcholine in the 1980s. Succinylcholine is considered the most common anesthetic for inducing MH, and dantrolene is an effective drug for treating MH. In the later period, the focus was on inducing anesthetic drugs such as halothane, and some MH animal models were also established to study pathogenesis. Since the 1990s, more research has focused on MH susceptibility, and the discovery of ryanodine receptors has given people a deeper understanding of MH at the genetic level. Afterward, the research topics focused on perioperative management, guideline development and patient safety. The keyword timeline co-occurrence visualization image is shown in supplementary Fig. 3.

**Fig. 7:**
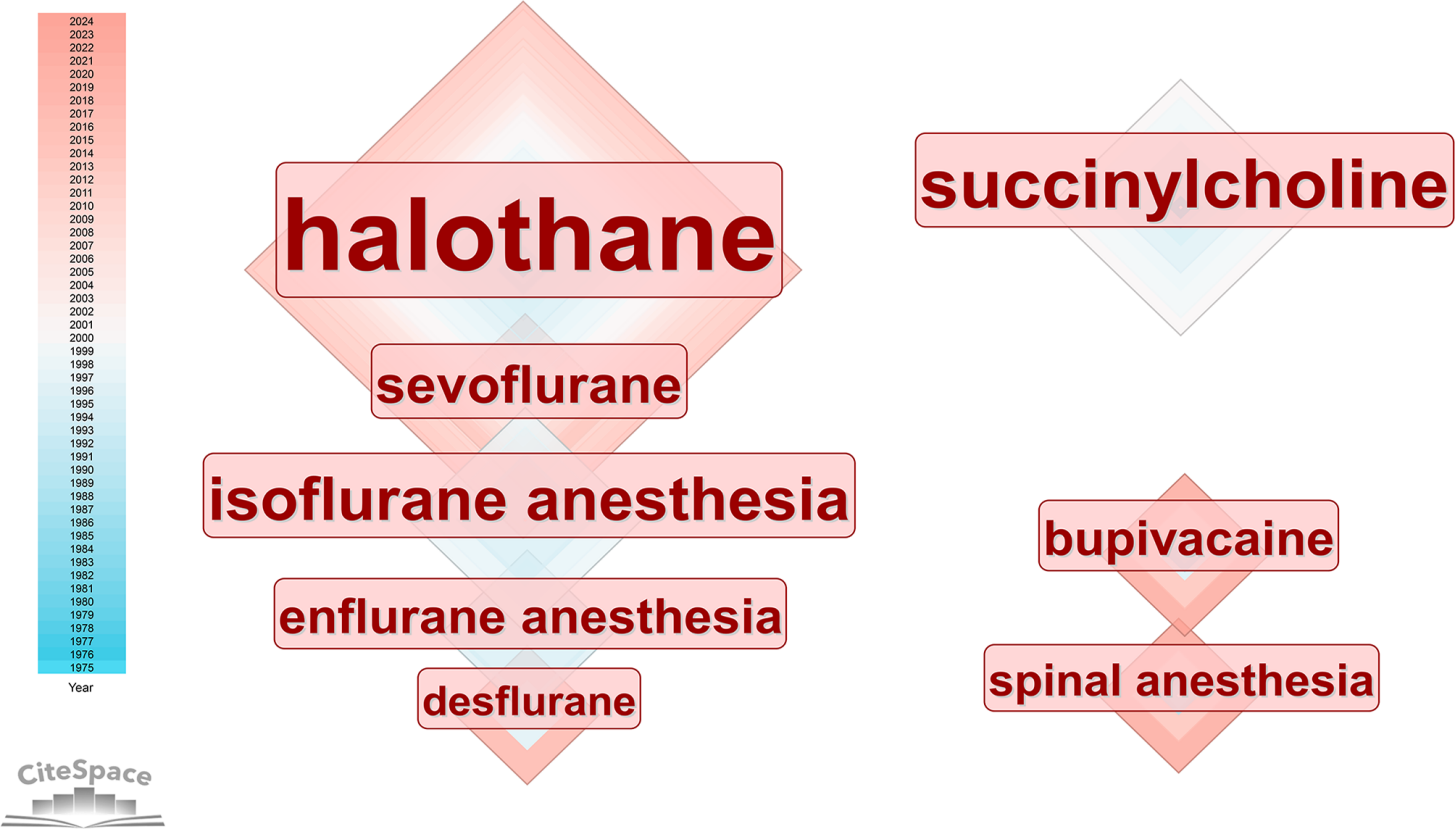
Visualization image of the keyword “anesthetic drugs”. The drugs that induce MH include inhaled anesthetics such as halothane, enflurane, desflurane, sevoflurane, and nondepolarizing anesthetics such as succinylcholine. Recently, local anesthetic bupivacaine and spinal anesthesia-induced MH have received increasing attention

**Fig. 8:**
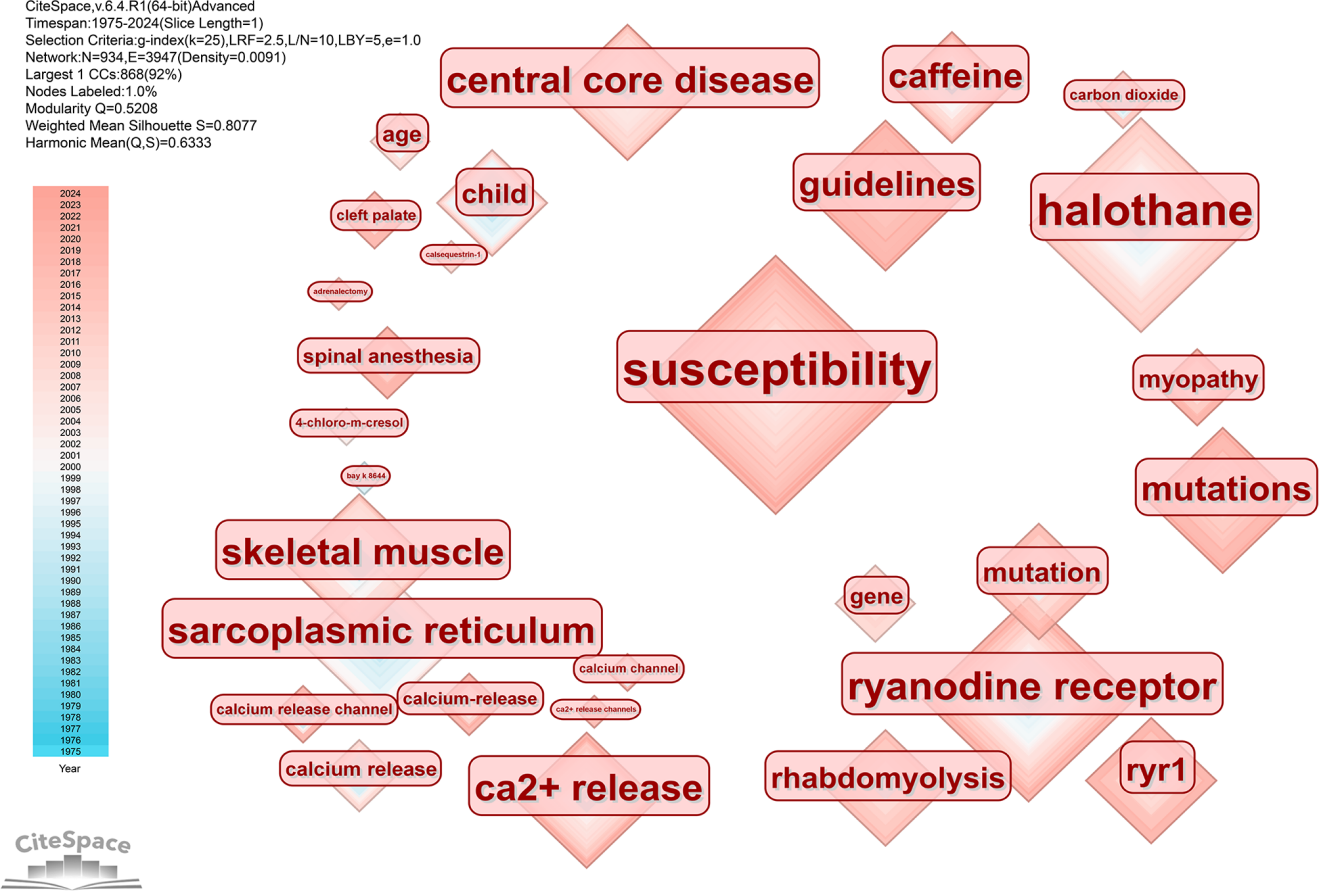
Keywords related to sensitivity research. Keywords related to sensitivity include the ryanodine receptor, skeletal muscle, Ca2 + release, etc

We extracted the anesthetic drugs that induce MH from the keywords. The drugs that induce MH include inhaled anesthetics such as halothane, enflurane, desflurane, sevoflurane, and nondepolarizing anesthetics such as succinylcholine. Recently, local anesthetic bupivacaine and spinal anesthesia-induced MH have received increasing attention. The keyword anesthetic drug visualization image is shown in Fig. 7. MH sensitivity is the key to early prediction of occurrence. Keywords related to sensitivity include *ryanodine receptor*, *skeletal muscle*, *Ca2 + release*, etc. The keywords related to sensitivity research are shown in Fig. 8.

## Discussion

In 2015, the Lancet Commission on Global Surgery raised the critical issue that 5 billion people worldwide lack access to safe and affordable surgical and anesthesia care. In recent years, nearly 200 million surgeries under general anesthesia have been performed worldwide. There is a large gap in medical safety between high-income countries and low-income countries [21]. With the use of dantrolene, the mortality rate of MH decrease from 80 to 0 -18.2% [22], whereas in low-income countries, it is still as high as 70% [2]. Early identification of sensitive populations, rapid diagnosis of MH, use of dantrolene, and multidisciplinary teamwork are vital for reducing mortality rates [1]. The incidence rate of MH is low, and medical personnel may lack knowledge of MH. Through training and communication, anesthesiologists and surgical nurses can improve their ability to identify MH. Expert consensus can also be established to ensure that medical personnel can quickly identify and handle MH.

From the keyword analysis, we found that *susceptibility* to malignant hyperthermia is the hottest subject of current research. Identifying susceptibility to MH is crucial for developing anesthesia management plans and can help reduce MH mortality rates [23]. The Ryr1 gene was sequenced and confirmed to encode calcium release from the sarcoplasmic reticulum of skeletal muscle. In 1991, Ryr1 mutations were shown to impair calcium homeostasis and increase susceptibility to MH [24]. In patients with Ryr1 mutations, halogenated volatile anesthetics or nonpolar skeletal muscle relaxants can cause Ryr1 to open, leading to uncontrolled Ca2^+^ release, increased sarcomere tension, and massive thermogenesis [25]. In vitro experiments have shown that primary skeletal muscle cells carrying R2509C-Ryr1 mutations exhibit dysfunctional Ca2^+^ dynamics [26]. In addition to regulating calcium release in skeletal muscle cells, Ryr1 also plays an important role in neuronal cells. Isoflurane induces a decrease in presynaptic cytoplasmic Ca2^+^ concentration and synaptic vesicle (SV) exocytosis in T4826I-Ryr1 mutant neurons. Ryr1 is a molecular target of the effect of isoflurane on presynaptic Ca^2+^ release. Most individuals susceptible to MH have defects in the Ryr1 gene on chromosome 19, and patients and their families suspected of MH reactions should undergo muscle contraction and genetic testing at specialized MH testing centers [1, 4].

**Fig. 9:**
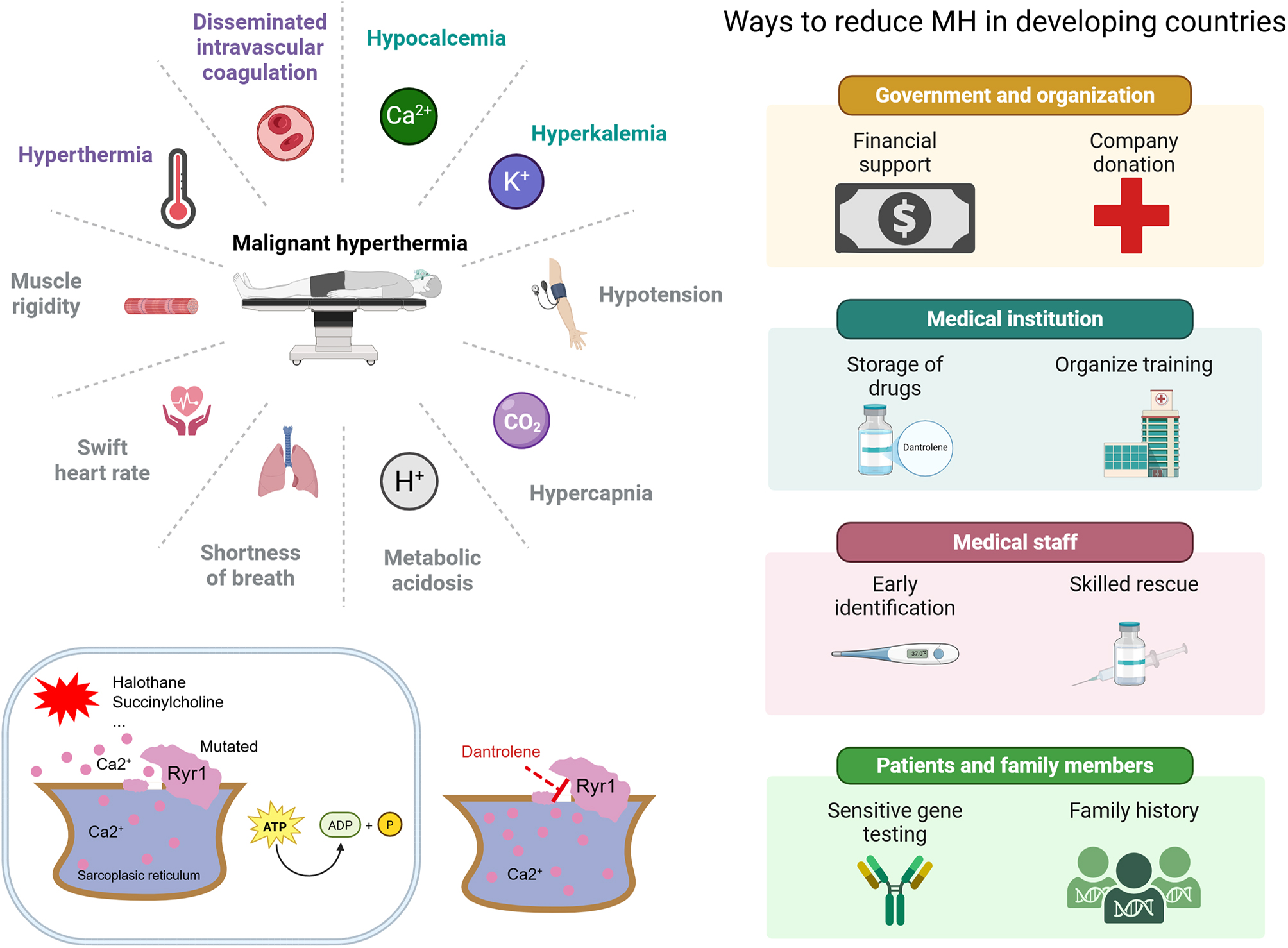
MH pathogenesis model diagram. MH is accompanied by various clinical symptoms. Early identification of MH and timely use of dantrolene, improving the MH rescue process, and strengthening regional cooperation are conducive to reducing MH mortality. The figure has been confirmed by Biorender with agreement number (FL27XTCGTF)

Dantrolene is a classic medicine for treating MH, and its use reduces the mortality rate from 80% to less than 10% [8]. However, dantrolene is expensive, and not every hospital has this drug in some developing countries. A nonprofit academic organization, namely, China Malignant Hyperthermia Emergency Assistance WeChat-based Group (CMHEA Group), collected data from 58 suspected cases of MH; only 14 patients (24.1%) received treatment with dantrolene, resulting in a mortality rate of 36.4%. There were 44 patients who did not receive dantrolene treatment, resulting in a mortality rate of 78.6% [27]. The situation of increased mortality due to drug shortages urgently needs to be improved. Due to the official introduction of dantrolene into China in October 2020, the shortage of dantrolene in China is expected to improve [28]. To provide insight into MH research from a developing country’s perspective, we present a brief overview of studies in China using the China National Knowledge Infrastructure (CNKI) database (see supplementary documents). In Brazil, telephone services have been used since 1991 to guide the diagnosis and treatment of MH-related complications [29]. In 2010, the European Malignant Hyperthermia Group (EMHG) developed guidelines for the management of MH [30]. The guidelines have undergone multiple rounds of updates, and the latest consensus was announced in September 2024 [31]. A key factor in the successful rescue of MH is the accessibility of the dantrolene. However, owing to the high price of dantrolene and the fact that MH is a rare disease with very low usage, many hospitals in developing countries do not have a budget to reserve dantrolene. A cross-sectional survey in China showed that only 8.1% of anesthesiologists reported having dantrolene in their hospital, and 5.6% expressed uncertainty [32]. The reserved dantrolene may not be used before expiration, resulting in hospital budget increase. Additionally, the inadequate drug supply chain and restrictions on drug registration have resulted in a limited supply of dantrolene in underdeveloped areas. Solving the problem of popularizing dantrolene requires cooperation from multiple parties. The government should encourage local pharmaceutical companies to produce generic drugs and reduce the price of dantrolene through multiple channels, such as government subsidies and international aid. The government needs to list dantrolene as an essential drug and ensure that more hospitals are equipped with dantrolene through funding and policy support. Cooperation with international organizations, such as the WHO, should be strengthened, and technology and drug donations should be enhanced. In addition, manufacture and market of inhaled anesthetics should develop strategic centers to provide training, simulation of MH cases, and provide dantrolene efficiently to hospitals by area of territory and population care. Furthermore, an efficient drug supply chain should be established to ensure that dantrolene can reach remote areas. The mechanism and key recommendations of MH are shown in Fig. 9.

### Limitations

Although our study analyzed the current status of MH, there were some limitations to be considered. First, we only searched three databases, WoS, PMC, and Scopus databases, but rare diseases were usually distributed across multiple platforms. Thus, any potentially missed articles were not included in the collection. Secondly, we only analyzed the abstract and keywords of the included articles. We did not analyze the main content of the articles, which may have resulted in missing some vital information. The literature types we included only include articles, not case reports, letters, or conference proceedings. Python can be used to customize the extraction of content in the future, which can be used across multiple platforms in advance and extract full-text information for analysis, thus obtaining more comprehensive data. Thirdly, although there are differences in MH mortality rates and publication numbers between advanced and developing economies, we do not have direct evidence to support the association between economic level and MH mortality rate. In the future, it is necessary to conduct research on MH health economics to explore the deep correlation between MH mortality rate and local economic development level, which will provide theoretical basis for future policy formulation.

## Conclusion

Owing to the lack of dantrolene and cooperation mechanisms in developing countries, the mortality rate caused by MH is still high. The government and pharmaceutical companies should collaborate to assist the availability and accessibility of dantrolene in developing countries. Early identification of MH, improvement of the MH rescue process, and strengthening of policy support are conducive to reducing MH mortality.

## Electronic supplementary material

Below is the link to the electronic supplementary material.

Supplementary Material 1

Supplementary Material 2

## Data Availability

The data that support the findings of this study are available on OneDrive at https://studentmust-my.sharepoint.com/:u:/g/personal/3230005647_student_must_edu_mo/EfDgjq0SnwFBsfNKUdSzOOwB6OI8nMV7J-Iw-WIPYFqhiw?e=fXWtGU.
