# Supplementary material for: Research hotspots and trends in malignant hyperthermia due to anesthesia from a global perspective: a bibliometric analysis from 1975 to 2024
Source: Orphanet J Rare Dis. 2025 Jul 1;20:325. doi: 10.1186/s13023-025-03766-5 (PMC12217809; doi:10.1186/s13023-025-03766-5)
Supplement: Supplementary file 1 — Supplementary Material 1 [file 13023_2025_3766_MOESM1_ESM.docx]

**Bibliometric analysis of the current status of research on malignant hyperthermia in China: based on the CNKI database**

To supplement the research trend of malignant hyperthermia in developing countries, we conducted further research using China as an example. To retrieve studies, we searched the China National Knowledge Infrastructure (CNKI) databases. Three hundred eighty-six journal articles were retrieved using malignant hyperthermia as the main topic, and 207 were ultimately included through reading abstracts. We conducted a brief analysis of the number of published articles, authors, and keywords.

Although developing countries face shortages in funding and medication, healthcare workers have also made significant efforts to reduce the mortality rate of MH by exploring treatment options that lack dantrolene. Fortunately, the diagnosis, treatment, and rescue system for malignant hyperthermia has gradually been established in China, and domestically produced single-agent drugs have also been developed. China has made significant progress in reducing the mortality rate of MH.

**Table 1** **The top 10 productive authors.**

| **Rank** | **Name** | **Number of publications** | |
| --- | --- | --- | --- |
| 1 | Xiang-Yang Guo | | 11 |
| 2 | Ai-Lun Luo | | 7 |
| 3 | Zhan-Gui Tang | | 5 |
| 4 | Miao-Ning Gu | | 4 |
| 5 | Yin-Lin Wang | | 4 |
| 6 | Hong Jiang | | 3 |
| 7 | Zai-Sheng Qin | | 3 |
| 8 | Zhong-Huang Xu | | 3 |
| 9 | Ming-Wen Ou-Yang | | 3 |
| 10 | Jin-Fang Xiao | | 3 |

**Table 2 Top 10 institutions with publications on MH from 1973 to 2024 in China**

| **Rank** | **Institution** | **Number of publications** |
| --- | --- | --- |
| 1 | Peking Union Medical College Hospital | 11 |
| 2 | Peking University Third Hospital | 5 |
| 3 | Southern Medical University Southern Hospital | 4 |
| 4 | The Ninth People's Hospital Affiliated to Shanghai Jiao Tong University School of Medicine | 4 |
| 5 | The Second Affiliated Hospital of Zhejiang University School of Medicine | 3 |
| 6 | Xiangya Hospital of Central South University | 3 |
| 7 | The First Affiliated Hospital of China Medical University | 3 |
| 8 | Tongji Hospital Affiliated to Tongji Medical College, Huazhong University of Science and Technology | 3 |
| 9 | Xiangya Stomatological Hospital of Central South University | 3 |
| 10 | General Armed Police Hospital | 2 |

Supplementary NCKI Fig 1. Annual publication trends. A total of 207 publications were included in CNKI study. The number of published articles reached a brief peak from 1995 to 2013.

Supplementary NCKI Fig 2. Author cooperation network. According to the author collaboration network, Ailun Luo, Xiangyang Guo, Zhonghuang Xu, and Yinglin Wang were the research groups with the highest number of publications. In addition, research groups centered around Miaoning Gu, Hong Jiang, and Zhangui Tang also played an important role.

Supplementary NCKI Fig 3. Keyword timeline co-occurrence visualization image. From the keyword timeline chart, it can be observed that research related to MH was rapidly developing from 1995 to 2014. In those years, research topics mainly focused on MH case reports and pathogenesis research. In recent years, MH research topics have mainly focused on MH training, expert consensus, and anesthesia management.
