# Supplementary material for: Research hotspots and trends in malignant hyperthermia due to anesthesia from a global perspective: a bibliometric analysis from 1975 to 2024
Source: Orphanet J Rare Dis. 2025 Jul 1;20:325. doi: 10.1186/s13023-025-03766-5 (PMC12217809; doi:10.1186/s13023-025-03766-5)
Supplement: Supplementary file 2 — Supplementary Material 2 [file 13023_2025_3766_MOESM2_ESM.docx]

**Supplementary figures**

**Supplementary Figure 1**

Supplementary Figure 1 Author cooperation network. Author visualization analysis revealed three main author networks that are relatively influential in this field. The three author networks are Denborough from Australian National University in Australia, Sessler from Univ Calif San Francisco in the United States, and Ellis from St James's University Hospital in the United Kingdom.

**Supplementary Figure 2**

Supplementary Figure 2 Institutions cooperation network. The most publications are from the University of California System (n=44, centrality=0.04).

**Supplementary Figure 3**

Supplementary Figure 3 Keyword timeline co-occurrence visualization image. The high-frequency keywords are MH (count=606, centrality=0.62), anesthesia (count=239, centrality=0.59), susceptibility (count=84, centrality=0.06), halothane (count=69, centrality=0.07), management (count=56, centrality=0.06), and dantrolene (count=48, centrality=0.07).
